# Supplementary material for: Comparison of a SARS-CoV-2 mRNA booster immunization containing additional antigens to a spike-based mRNA vaccine against Omicron BA.5 infection in hACE2 mice
Source: PLoS One. 2024 Dec 3;19(12):e0314061. doi: 10.1371/journal.pone.0314061 (PMC11614295; doi:10.1371/journal.pone.0314061)
Supplement: S1 File — (DOCX) [file pone.0314061.s001.docx]

S1 Table. Individual mouse ELISPOT data for IFN-γ and IL-4 responses to SARS-CoV-2 beta spike protein

| **Assay** | **Matrix** | **Treatment** | **Dose (µg)** | **Study Day** | **Animal No.** | **Units** | **Data** |
| --- | --- | --- | --- | --- | --- | --- | --- |
| ELISPOT | IFN-γ  IL-4 | Spike-B.1.351 | 10 µg x2 | Day 42 | 1a | SFU per  2.5 X 10^5^ cells | **IFN-γ :** 608  **IL-4 :** 14 |
| ELISPOT | IFN-γ  IL-4 | Spike-B.1.351 | 10 µg x2 | Day 42 | 1b | SFU per  2.5 X 10^5^ cells | **IFN-γ :** 1042.5  **IL-4 :** 44 |
| ELISPOT | IFN-γ  IL-4 | Spike-B.1.351 | 10 µg x2 | Day 42 | 2a | SFU per  2.5 X 10^5^ cells | **IFN-γ :** 1740  **IL-4 :** 25 |
| ELISPOT | IFN-γ  IL-4 | Spike-B.1.351 | 10 µg x2 | Day 42 | 2b | SFU per  2.5 X 10^5^ cells | **IFN-γ :** 588  **IL-4 :** 16 |
| ELISPOT | IFN-γ  IL-4 | Spike-B.1.351 | 10 µg x2 | Day 42 | 3a | SFU per  2.5 X 10^5^ cells | **IFN-γ :** 909.5  **IL-4 :** 32.5 |
| ELISPOT | IFN-γ  IL-4 | Spike-B.1.351 | 10 µg x2 | Day 42 | 3b | SFU per  2.5 X 10^5^ cells | **IFN-γ :** 144.5  **IL-4 :** 14.5 |
| ELISPOT | IFN-γ  IL-4 | Control | 10 µg x2 | Day 42 | 6a | SFU per  2.5 X 10^5^ cells | **IFN-γ :** 10.5  **IL-4 :** 14 |
| ELISPOT | IFN-γ  IL-4 | Control | 10 µg x2 | Day 42 | 6b | SFU per  2.5 X 10^5^ cells | **IFN-γ :** 5  **IL-4 :** 21.5 |
| ELISPOT | IFN-γ  IL-4 | Control | 10 µg x2 | Day 42 | 7a | SFU per  2.5 X 10^5^ cells | **IFN-γ :** 8  **IL-4 :** 20 |
| ELISPOT | IFN-γ  IL-4 | Control | 10 µg x2 | Day 42 | 7b | SFU per  2.5 X 10^5^ cells | **IFN-γ :** 2.5  **IL-4 :** 4.5 |

S2 Table. Flow cytometry data from SARS-CoV-2 beta spike peptide pool stimulations using mouse spleens.

| **Treatment** | **Dose (µg)** | **Study Day** | **Animal No.** | **Splenocyte populations (%) following**  **Beta spike peptide pool stimulations** | |
| --- | --- | --- | --- | --- | --- |
|  |  |  |  | **CD4^+^** | **CD8^+^** |
| Spike-B.1.351 *(IMx2)* | 10 µg x2 | Day 42 | 1a | **IFN-γ:** 0.270  **TNFα:** 1.14  **IL-2:** 0.259  **IL-4:** 0.060  **IL-5:** 0.0 | **IFN-γ:** 3.880  **TNFα:** 2.181  **IL-2:** 0.13  **IL-4:** 0.18  **IL-5:** 0.0 |
| Spike-B.1.351 *(IMx2)* | 10 µg x2 | Day 42 | 1b | **IFN-γ:** 0.251  **TNFα:** 0.39176  **IL-2:** 0.242  **IL-4:** 0.0  **IL-5:** 0.16 | **IFN-γ:** 2.617  **TNFα:** 0.962  **IL-2:** 0.08  **IL-4:** 0.0  **IL-5:** 0.14 |
| Spike-B.1.351 *(IMx2)* | 10 µg x2 | Day 42 | 2a | **IFN-γ:** 0.336  **TNFα:** 0.553  **IL-2:** 0.160  **IL-4:** 0.0  **IL-5:** 0.0 | **IFN-γ:** 4.230  **TNFα:** 1.870  **IL-2:** 0.004  **IL-4:** 0.0  **IL-5:** 0.0 |
| Spike-B.1.351 *(IMx2)* | 10 µg x2 | Day 42 | 2b | **IFN-γ:** 0.222  **TNFα:** 0.371  **IL-2:** 0.145  **IL-4:** 0.0  **IL-5:** 0.0 | **IFN-γ:** 1.302  **TNFα:** 0.611  **IL-2:** 0.04  **IL-4:** 0.0  **IL-5:** 0.0 |
| Spike-B.1.351 *(IMx2)* | 10 µg x2 | Day 42 | 3a | **IFN-γ:** 0.686  **TNFα:** 0.820  **IL-2:** 0.346  **IL-4:** 0.020  **IL-5:** 0.0 | **IFN-γ:** 3.307  **TNFα:** 1.300  **IL-2:** 0.06  **IL-4:** 0.0  **IL-5:** 0.00.0 |
| Spike-B.1.351 *(IMx2)* | 10 µg x2 | Day 42 | 3b | **IFN-γ:** 0.186  **TNFα:** 0.292  **IL-2:** 0.110  **IL-4:** 0.0  **IL-5:** 0.0 | **IFN-γ:** 0.883  **TNFα:** 0.411  **IL-2:** 0.0  **IL-4:** 0.0  **IL-5:** 0.03 |
| Buffer only *(IMx2)* | 10 µg x2 | Day 42 | 6a | **IFN-γ:** 0.081  **TNFα:** 0.0  **IL-2:** 0.002  **IL-4:** 0.0  **IL-5:** 0.135 | **IFN-γ:** 0.069  **TNFα:** 0.0  **IL-2:** 0.064  **IL-4:** 0.008  **IL-5:** 0.0 |
| Buffer only *(IMx2)* | 10 µg x2 | Day 42 | 6b | **IFN-γ:** 0.011  **TNFα:** 0.0  **IL-2:** 0.016  **IL-4:** 0.0  **IL-5:** 0.0 | **IFN-γ:** 0.005  **TNFα:** 0.069  **IL-2:** 0.0  **IL-4:** 0.0  **IL-5:** 0.0 |
| Buffer only *(IMx2)* | 10 µg x2 | Day 42 | 7a | **IFN-γ:** 0.025  **TNFα:** 0.0  **IL-2:** 0.0  **IL-4:** 0.01  **IL-5:** 0.20 | **IFN-γ:** 0.0  **TNFα:** 0.035  **IL-2:** 0.0  **IL-4:** 0.0  **IL-5:** 0.07 |
| Buffer only *(IMx2)* | 10 µg x2 | Day 42 | 7b | **IFN-γ:** 0.014  **TNFα:** 0.014  **IL-2:** 0.0  **IL-4:** 0.14  **IL-5:** 0.050 | **IFN-γ:** 0.0  **TNFα:** 0.0  **IL-2:** 0.0  **IL-4:** 0.091  **IL-5:** 0.0 |

**S3 Table. Individual mouse serum neutralization titers at 3 weeks following the second immunization**

| **Treatment** | **Animal**  **No.** | **Mouse sera ID_50_ Titers** | | | | | | |
| --- | --- | --- | --- | --- | --- | --- | --- | --- |
|  |  | **Wuhan-Hu-1**  **(D614G)** | **Beta B.1.351** | **BA.1** | **BA.2** | **BA.2.12.1** | **BA.5** | **XBB1.5** |
| Spike-B.1.351 *(IMx2)* | 1a | 10422.8866 | 40876.87 | 162.7141 | 162.7141 | 1 | 1 | 1 |
| Spike-B.1.351 *(IMx2)* | 1b | 31973.8509 | 39925.04 | 1554.42 | 313.1793 | 151.5934 | 151.5934 | 1 |
| Spike-B.1.351 *(IMx2)* | 2a | 11988.5217 | 56169.23 | 3457.868 | 2459.168 | 325.8664 | 125.8664 | 1 |
| Spike-B.1.351 *(IMx2)* | 2b | 2645.67991 | 18805.92 | 435.4982 | 255.4669 | 155.4669 | 1 | 1 |
| Spike-B.1.351 *(IMx2)* | 3a | 118646.298 | 254904.2 | 570.5818 | 304.7527 | 1502.174 | 50.44274 | 50.442 |
| Spike-B.1.351 *(IMx2)* | 3b | 19652.6654 | 32949.94 | 2165.133 | 1256.853 | 1256.85 | 175.7975 | 1 |
| Buffer only *(IMx2)* | 6a | 1 | 1 | 1 | 1 | 1 | 1 | 1 |
| Buffer only *(IMx2)* | 6b | 1 | 1 | 1 | 1 | 1 | 1 | 1 |
| Buffer only *(IMx2)* | 7a | 1 | 1 | 1 | 1 | 1 | 1 | 1 |
| Buffer only *(IMx2)* | 7b | 1 | 1 | 1 | 1 | 1 | 1 | 1 |

**S4 Table. Individual mouse serum neutralization titers at 54 days following the second immunization**

| **Treatment** | **Animal**  **No.** | **Mouse sera ID_50_ Titers** | | | | | | |
| --- | --- | --- | --- | --- | --- | --- | --- | --- |
|  |  | **Wuhan-Hu-1**  **(D614G)** | **Beta B.1.351** | **BA.1** | **BA.2** | **BA.2.12.1** | **BA.5** | **XBB1.5** |
| Spike-B.1.351 *(IMx3)* | 65228 | 6370.94747 | 16670.68 | 2714.376 | 3111.282 | 4036.466 | 242.657 | 1 |
| Spike-B.1.351 *(IMx3)* | 65230 | 14397.029 | 20651.36 | 707.2676 | 2765.765 | 9438.12 | 712.1944 | 1 |
| Spike-B.1.351 *(IMx3)* | 65232 | 87969.5173 | 68325.79 | 5453.575 | 2263.988 | 2971.715 | 864.8772 | 1 |
| Spike-B.1.351 *(IMx3)* | 65233 | 4974.85885 | 10712.25 | N.D. | N.D. | 1 | 962.66 | 1 |
| Spike-B.1.351 *(IMx2)* +  Spike-B.1.351-NM *(IM)* | 65249 | 44159.2831 | 30134.17 | 1703.335 | 1767.168 | 1259.863 | 751.5531 | 131.177 |
| Spike-B.1.351 *(IMx2)* +  Spike-B.1.351-NM *(IM)* | 65250 | 35256.3886 | 60350.74 | 2773.867 | 3920.141 | 1523.635 | 1364.576 | 1 |
| Spike-B.1.351 *(IMx2)* +  Spike-B.1.351-NM *(IM)* | 65251 | 67914.5957 | 170779.1 | 2443.322 | 1816.437 | 1194.572 | 925.772 | 1 |
| Spike-B.1.351 *(IMx2)* +  Spike-B.1.351-NM *(IM)* | 65252 | 22030.2856 | 39152.75 | 1438.972 | 684.7901 | 940.6962 | 337.5456 | 1 |
| Buffer only *(IMx2)* | 65242 | 1 | 1 | 1 | 1 | 1 | 1 | 1 |
| Buffer only *(IMx2)* | 65243 | 1 | 1 | 1 | 1 | 1 | 1 | 1 |
| Buffer only *(IMx2)* | 65244 | 1 | 1 | 1 | 1 | 1 | 1 | 1 |
| Buffer only *(IMx2)* | 65245 | 1 | 1 | 1 | 1 | 1 | 1 | 1 |

**S5 Table. Individual mouse weight loss following SARS-CoV-2 BA.5 infection.**

| **Treatment** | **Animal No.** | **Mouse Weights**  **Weight loss (%)** | | | | |
| --- | --- | --- | --- | --- | --- | --- |
|  |  | **Day 1** | **Day 2** | **Day 3** | **Day 4** | **Day 5** |
| Spike-B.1.351 *(IMx3)* | 65228 | -8.646616541 | -12.78195489 | -10.90225564 | -18.42105263 | -20.30075188 |
| Spike-B.1.351 *(IMx3)* | 65230 | -8.558558559 | -12.16216216 | -11.26126126 | -16.66666667 | -9.009009009 |
| Spike-B.1.351 *(IMx3)* | 65232 | -0.819672131 | -7.786885246 | -3.68852459 | -4.918032787 | -4.098360656 |
| Spike-B.1.351 *(IMx3)* | 65233 | -3.734439834 | -7.883817427 | -11.61825726 | -12.86307054 | 4.564315353 |
| Spike-B.1.351 *(IMx3)* | 65234 | -1.449275362 | -2.536231884 | -1.811594203 | -0.724637681 | -1.449275362 |
| Spike-B.1.351 *(IMx3)* | 65235 | -5.217391304 | -11.5942029 | -16.8115942 | -20.28985507 | -19.71014493 |
| Spike-B.1.351 *(IMx2)* +  Spike-B.1.351-NM *(IM)* | 65249 | -6.79245283 | -10.56603774 | -10.94339623 | -17.35849057 | -16.22641509 |
| Spike-B.1.351 *(IMx2)* +  Spike-B.1.351-NM *(IM)* | 65250 | -4.210526316 | -5.614035088 | -6.315789474 | -14.03508772 | -8.771929825 |
| Spike-B.1.351 *(IMx2)* +  Spike-B.1.351-NM *(IM)* | 65251 | -4.301075269 | -10.75268817 | -8.243727599 | -16.84587814 | -15.77060932 |
| Spike-B.1.351 *(IMx2)* +  Spike-B.1.351-NM *(IM)* | 65252 | -11.55555556 | -17.77777778 | -20 | -24.44444444 | -33.77777778 |
| Spike-B.1.351 *(IMx2)* +  Spike-B.1.351-NM *(IM)* | 65253 | -7.766990291 | -14.5631068 | -16.01941748 | -22.81553398 | -8.252427184 |
| Spike-B.1.351 *(IMx2)* +  Spike-B.1.351-NM *(IM)* | 65254 | -7.906976744 | -13.95348837 | -17.20930233 | -24.18604651 | -20.93023256 |
| Spike-B.1.351 *(IMx2)* +  Spike-B.1.351-NM *(IM)* | 65255 | -4.016064257 | -9.638554217 | -9.638554217 | -16.46586345 | -12.85140562 |
| Spike-B.1.351 *(IMx2)* +  Spike-B.1.351-NM *(IM)* | 65256 | -3.68852459 | -11.06557377 | -18.85245902 | -24.18032787 | -21.72131148 |
| Buffer only *(IMx3)* | 65242 | 0.448430493 | -9.417040359 | -11.21076233 | -17.04035874 | -19.28251121 |
| Buffer only *(IMx3)* | 65243 | -1.70212766 | -4.255319149 | -10.63829787 | -16.59574468 | -19.57446809 |
| Buffer only *(IMx3)* | 65244 | -0.81300813 | -6.504065041 | -10.56910569 | -16.2601626 | -22.35772358 |
| Buffer only *(IMx3)* | 65245 | -2.523659306 | -8.201892744 | -9.148264984 | -14.82649842 | -19.55835962 |

**S6 Table. Individual mouse clinical scores following SARS-CoV-2 BA.5 infection.**

| **Treatment** | **Animal No.** | **Mouse Weights**  **Weight loss (%)** | | | | |
| --- | --- | --- | --- | --- | --- | --- |
|  |  | **Day 1** | **Day 2** | **Day 3** | **Day 4** | **Day 5** |
| Spike-B.1.351 *(IMx3)* | 65228 | 2 | 2 | 2 | 2 | 3 |
| Spike-B.1.351 *(IMx3)* | 65230 | 2 | 2 | 2 | 2 | 2 |
| Spike-B.1.351 *(IMx3)* | 65232 | 1 | 2 | 1 | 1 | 1 |
| Spike-B.1.351 *(IMx3)* | 65233 | 1 | 2 | 3 | 3 | 1 |
| Spike-B.1.351 *(IMx3)* | 65234 | 1 | 1 | 2 | 2 | 1 |
| Spike-B.1.351 *(IMx3)* | 65235 | 2 | 2 | 3 | 4 | 2 |
| Spike-B.1.351 *(IMx2)* +  Spike-B.1.351-NM *(IM)* | 65249 | 2 | 2 | 2 | 2 | 2 |
| Spike-B.1.351 *(IMx2)* +  Spike-B.1.351-NM *(IM)* | 65250 | 1 | 2 | 2 | 2 | 2 |
| Spike-B.1.351 *(IMx2)* +  Spike-B.1.351-NM *(IM)* | 65251 | 1 | 2 | 2 | 2 | 2 |
| Spike-B.1.351 *(IMx2)* +  Spike-B.1.351-NM *(IM)* | 65252 | 2 | 2 | 2 | 3 | 3 |
| Spike-B.1.351 *(IMx2)* +  Spike-B.1.351-NM *(IM)* | 65253 | 2 | 2 | 2 | 4 | 2 |
| Spike-B.1.351 *(IMx2)* +  Spike-B.1.351-NM *(IM)* | 65254 | 2 | 2 | 2 | 3 | 3 |
| Spike-B.1.351 *(IMx2)* +  Spike-B.1.351-NM *(IM)* | 65255 | 1 | 3 | 2 | 2 | 2 |
| Spike-B.1.351 *(IMx2)* +  Spike-B.1.351-NM *(IM)* | 65256 | 1 | 3 | 2 | 3 | 3 |
| Buffer only *(IMx3)* | 65242 | 1 | 2 | 2 | 2 | 2 |
| Buffer only *(IMx3)* | 65243 | 1 | 1 | 2 | 2 | 2 |
| Buffer only *(IMx3)* | 65244 | 1 | 2 | 2 | 2 | 3 |
| Buffer only *(IMx3)* | 65245 | 1 | 2 | 2 | 2 | 2 |

S7 Table. Amount of infectious virus in oral swabs of mice 1-day post-intranasal infection with SARS-CoV-2 BA.5

| **Assay** | **Matrix** | **Treatment** | **Study Day** | **Animal No.** | **Units** | **Data** |
| --- | --- | --- | --- | --- | --- | --- |
| TCID_50_ | CPE of Vero cells | Spike-B.1.351*(IMx3)* | Day 134 | 65228 | TCID_50_ | 6.32E+00 |
| TCID_50_ | CPE of Vero cells | Spike-B.1.351*(IMx3)* | Day 134 | 65230 | TCID_50_ | 6.32E+00 |
| TCID_50_ | CPE of Vero cells | Spike-B.1.351*(IMx3)* | Day 134 | 65232 | TCID_50_ | 6.32E+00 |
| TCID_50_ | CPE of Vero cells | Spike-B.1.351*(IMx3)* | Day 134 | 65233 | TCID_50_ | 6.32E+00 |
| TCID_50_ | CPE of Vero cells | Spike-B.1.351*(IMx3)* | Day 134 | 65234 | TCID_50_ | 6.32E+00 |
| TCID_50_ | CPE of Vero cells | Spike-B.1.351*(IMx3)* | Day 134 | 65235 | TCID_50_ | 6.32E+00 |
| TCID_50_ | CPE of Vero cells | Spike-B.1.351 *(IMx2)* +  Spike-B.1.351-NM *(IM)* | Day 134 | 65249 | TCID_50_ | 6.32E+00 |
| TCID_50_ | CPE of Vero cells | Spike-B.1.351 *(IMx2)* +  Spike-B.1.351-NM *(IM)* | Day 134 | 65250 | TCID_50_ | 6.32E+00 |
| TCID_50_ | CPE of Vero cells | Spike-B.1.351 *(IMx2)* +  Spike-B.1.351-NM *(IM))* | Day 134 | 65251 | TCID_50_ | 6.32E+00 |
| TCID_50_ | CPE of Vero cells | Spike-B.1.351 *(IMx2)* +  Spike-B.1.351-NM *(IM)* | Day 134 | 65252 | TCID_50_ | 1.36E+01 |
| TCID_50_ | CPE of Vero cells | Spike-B.1.351 *(IMx2)* +  Spike-B.1.351-NM *(IM)* | Day 134 | 65253 | TCID_50_ | 6.32E+00 |
| TCID_50_ | CPE of Vero cells | Spike-B.1.351 *(IMx2)* +  Spike-B.1.351-NM *(IM)* | Day 134 | 65254 | TCID_50_ | 2.94E+01 |
| TCID_50_ | CPE of Vero cells | Spike-B.1.351 *(IMx2)* +  Spike-B.1.351-NM *(IM)* | Day 134 | 65255 | TCID_50_ | 6.32E+00 |
| TCID_50_ | CPE of Vero cells | Spike-B.1.351 *(IMx2)* +  Spike-B.1.351-NM *(IM)* | Day 134 | 65256 | TCID_50_ | 1.36E+01 |
| TCID_50_ | CPE of Vero cells | Buffer only *(IMx3)* | Day 134 | 65242 | TCID_50_ | 6.32E+02 |
| TCID_50_ | CPE of Vero cells | Buffer only *(IMx3)* | Day 134 | 65243 | TCID_50_ | 6.32E+01 |
| TCID_50_ | CPE of Vero cells | Buffer only *(IMx3)* | Day 134 | 65244 | TCID_50_ | 2.94E+03 |
| TCID_50_ | CPE of Vero cells | Buffer only *(IMx3)* | Day 134 | 65245 | TCID_50_ | 1.36E+03 |

S8 Table. Amount of SARS-CoV-2 viral RNA in oral swabs of mice 1-day post-intranasal infection with SARS-CoV-2 BA.5

| **Assay** | **Matrix** | **Treatment** | **Study Day** | **Animal No.** | **Units** | **Data** |
| --- | --- | --- | --- | --- | --- | --- |
| qPCR | Ct value | Spike-B.1.351*(IMx3)* | Day 134 | 65228 | RNA copies | 75957.4348 |
| qPCR | Ct value | Spike-B.1.351*(IMx3)* | Day 134 | 65230 | RNA copies | 287271.4787 |
| qPCR | Ct value | Spike-B.1.351*(IMx3)* | Day 134 | 65232 | RNA copies | 15178.27226 |
| qPCR | Ct value | Spike-B.1.351*(IMx3)* | Day 134 | 65233 | RNA copies | 53522.71388 |
| qPCR | Ct value | Spike-B.1.351*(IMx3)* | Day 134 | 65234 | RNA copies | 34621.23416 |
| qPCR | Ct value | Spike-B.1.351*(IMx3)* | Day 134 | 65235 | RNA copies | 17595.98959 |
| qPCR | Ct value | Spike-B.1.351 *(IMx2)* +  Spike-B.1.351-NM *(IM)* | Day 134 | 65249 | RNA copies | 222229.7803 |
| qPCR | Ct value | Spike-B.1.351 *(IMx2)* +  Spike-B.1.351-NM *(IM)* | Day 134 | 65250 | RNA copies | 56518.10265 |
| qPCR | Ct value | Spike-B.1.351 *(IMx2)* +  Spike-B.1.351-NM *(IM)* | Day 134 | 65251 | RNA copies | 41083.68406 |
| qPCR | Ct value | Spike-B.1.351 *(IMx2)* +  Spike-B.1.351-NM *(IM)* | Day 134 | 65252 | RNA copies | 287271.4787 |
| qPCR | Ct value | Spike-B.1.351 *(IMx2)* +  Spike-B.1.351-NM *(IM)* | Day 134 | 65253 | RNA copies | 66548.13926 |
| qPCR | Ct value | Spike-B.1.351 *(IMx2)* +  Spike-B.1.351-NM *(IM)* | Day 134 | 65254 | RNA copies | 665533.1642 |
| qPCR | Ct value | Spike-B.1.351 *(IMx2)* +  Spike-B.1.351-NM *(IM)* | Day 134 | 65255 | RNA copies | 77750.96127 |
| qPCR | Ct value | Spike-B.1.351 *(IMx2)* +  Spike-B.1.351-NM *(IM)* | Day 134 | 65256 | RNA copies | 383086.3988 |
| qPCR | Ct value | Buffer only *(IMx3)* | Day 134 | 65242 | RNA copies | 1773616.657 |
| qPCR | Ct value | Buffer only *(IMx3)* | Day 134 | 65243 | RNA copies | 4914080.634 |
| qPCR | Ct value | Buffer only *(IMx3)* | Day 134 | 65244 | RNA copies | 17600096.59 |
| qPCR | Ct value | Buffer only *(IMx3)* | Day 134 | 65245 | RNA copies | 16410001.42 |

S9 Table. Amount of infectious virus in oral swabs of mice 3 days post-intranasal infection with SARS-CoV-2 BA.5

| **Assay** | **Matrix** | **Treatment** | **Study Day** | **Animal No.** | **Units** | **Data** |
| --- | --- | --- | --- | --- | --- | --- |
| TCID_50_ | CPE of Vero cells | Spike-B.1.351*(IMx3)* | Day 136 | 65228 | TCID_50_ | 6.32E+00 |
| TCID_50_ | CPE of Vero cells | Spike-B.1.351*(IMx3)* | Day 136 | 65230 | TCID_50_ | 6.32E+00 |
| TCID_50_ | CPE of Vero cells | Spike-B.1.351*(IMx3)* | Day 136 | 65232 | TCID_50_ | 6.32E+00 |
| TCID_50_ | CPE of Vero cells | Spike-B.1.351*(IMx3)* | Day 136 | 65233 | TCID_50_ | 6.32E+00 |
| TCID_50_ | CPE of Vero cells | Spike-B.1.351*(IMx3)* | Day 136 | 65234 | TCID_50_ | 6.32E+00 |
| TCID_50_ | CPE of Vero cells | Spike-B.1.351*(IMx3)* | Day 136 | 65235 | TCID_50_ | 6.32E+00 |
| TCID_50_ | CPE of Vero cells | Spike-B.1.351 *(IMx2)* +  Spike-B.1.351-NM *(IM)* | Day 136 | 65249 | TCID_50_ | 6.32E+00 |
| TCID_50_ | CPE of Vero cells | Spike-B.1.351 *(IMx2)* +  Spike-B.1.351-NM *(IM)* | Day 136 | 65250 | TCID_50_ | 6.32E+00 |
| TCID_50_ | CPE of Vero cells | Spike-B.1.351 *(IMx2)* +  Spike-B.1.351-NM *(IM)* | Day 136 | 65251 | TCID_50_ | 6.32E+00 |
| TCID_50_ | CPE of Vero cells | Spike-B.1.351 *(IMx2)* +  Spike-B.1.351-NM *(IM)* | Day 136 | 65252 | TCID_50_ | 6.32E+00 |
| TCID_50_ | CPE of Vero cells | Spike-B.1.351 *(IMx2)* +  Spike-B.1.351-NM *(IM)* | Day 136 | 65253 | TCID_50_ | 6.32E+00 |
| TCID_50_ | CPE of Vero cells | Spike-B.1.351 *(IMx2)* +  Spike-B.1.351-NM *(IM)* | Day 136 | 65254 | TCID_50_ | 6.32E+00 |
| TCID_50_ | CPE of Vero cells | Spike-B.1.351 *(IMx2)* +  Spike-B.1.351-NM *(IM)* | Day 136 | 65255 | TCID_50_ | 6.32E+00 |
| TCID_50_ | CPE of Vero cells | Spike-B.1.351 *(IMx2)* +  Spike-B.1.351-NM *(IM)* | Day 136 | 65256 | TCID_50_ | 6.32E+00 |
| TCID_50_ | CPE of Vero cells | Buffer only *(IMx3)* | Day 136 | 65242 | TCID_50_ | 6.32E+00 |
| TCID_50_ | CPE of Vero cells | Buffer only *(IMx3)* | Day 136 | 65243 | TCID_50_ | 6.32E+00 |
| TCID_50_ | CPE of Vero cells | Buffer only *(IMx3)* | Day 136 | 65244 | TCID_50_ | 6.32E+00 |
| TCID_50_ | CPE of Vero cells | Buffer only *(IMx3)* | Day 136 | 65245 | TCID_50_ | 1.36E+01 |

S10 Table. Amount of SARS-CoV-2 viral RNA in oral swabs of mice 3 days post-intranasal infection with SARS-CoV-2 BA.5

| **Assay** | **Matrix** | **Treatment** | **Study Day** | **Animal No.** | **Units** | **Data** |
| --- | --- | --- | --- | --- | --- | --- |
| qPCR | Ct value | Spike-B.1.351*(IMx3)* | Day 136 | 65228 | RNA copies | 0 |
| qPCR | Ct value | Spike-B.1.351*(IMx3)* | Day 136 | 65230 | RNA copies | 85.3387589 |
| qPCR | Ct value | Spike-B.1.351*(IMx3)* | Day 136 | 65232 | RNA copies | 57.83912266 |
| qPCR | Ct value | Spike-B.1.351*(IMx3)* | Day 136 | 65233 | RNA copies | 0 |
| qPCR | Ct value | Spike-B.1.351*(IMx3)* | Day 136 | 65234 | RNA copies | 251.6267349 |
| qPCR | Ct value | Spike-B.1.351*(IMx3)* | Day 136 | 65235 | RNA copies | 46529.16523 |
| qPCR | Ct value | Spike-B.1.351 *(IMx2)* +  Spike-B.1.351-NM *(IM)* | Day 136 | 65249 | RNA copies | 368.3858291 |
| qPCR | Ct value | Spike-B.1.351 *(IMx2)* +  Spike-B.1.351-NM *(IM)* | Day 136 | 65250 | RNA copies | 3435.027893 |
| qPCR | Ct value | Spike-B.1.351 *(IMx2)* +  Spike-B.1.351-NM *(IM)* | Day 136 | 65251 | RNA copies | 4688.889181 |
| qPCR | Ct value | Spike-B.1.351 *(IMx2)* +  Spike-B.1.351-NM *(IM)* | Day 136 | 65252 | RNA copies | 3571.27037 |
| qPCR | Ct value | Spike-B.1.351 *(IMx2)* +  Spike-B.1.351-NM *(IM)* | Day 136 | 65253 | RNA copies | 7192.619018 |
| qPCR | Ct value | Spike-B.1.351 *(IMx2)* +  Spike-B.1.351-NM *(IM)* | Day 136 | 65254 | RNA copies | 242.0272798 |
| qPCR | Ct value | Spike-B.1.351 *(IMx2)* +  Spike-B.1.351-NM *(IM)* | Day 136 | 65255 | RNA copies | 3571.27037 |
| qPCR | Ct value | Spike-B.1.351 *(IMx2)* +  Spike-B.1.351-NM *(IM)* | Day 136 | 65256 | RNA copies | 78970.11167 |
| qPCR | Ct value | Buffer only *(IMx3)* | Day 136 | 65242 | RNA copies | 3228521.498 |
| qPCR | Ct value | Buffer only *(IMx3)* | Day 136 | 65243 | RNA copies | 635182.8251 |
| qPCR | Ct value | Buffer only *(IMx3)* | Day 136 | 65244 | RNA copies | 255631.9437 |
| qPCR | Ct value | Buffer only *(IMx3)* | Day 136 | 65245 | RNA copies | 569639.1784 |

S11 Table. Amount of infectious virus in lung tissues of mice 5 days post-intranasal infection with SARS-CoV-2 BA.5

| **Assay** | **Matrix** | **Treatment** | **Study Day** | **Animal No.** | **Units** | **Data** |
| --- | --- | --- | --- | --- | --- | --- |
| TCID_50_ | CPE of Vero cells | Spike-B.1.351*(IMx3)* | Day 138 | 65228 | TCID_50_**/**  100 mg tissue | 795.7124842 |
| TCID_50_ | CPE of Vero cells | Spike-B.1.351*(IMx3)* | Day 138 | 65230 | TCID_50_**/**  100 mg tissue | 806.9053708 |
| TCID_50_ | CPE of Vero cells | Spike-B.1.351*(IMx3)* | Day 138 | 65232 | TCID_50_**/**  100 mg tissue | 528.0334728 |
| TCID_50_ | CPE of Vero cells | Spike-B.1.351*(IMx3)* | Day 138 | 65233 | TCID_50_**/**  100 mg tissue | 964.8318043 |
| TCID_50_ | CPE of Vero cells | Spike-B.1.351*(IMx3)* | Day 138 | 65234 | TCID_50_**/**  100 mg tissue | 390.2288188 |
| TCID_50_ | CPE of Vero cells | Spike-B.1.351*(IMx3)* | Day 138 | 65235 | TCID_50_**/**  100 mg tissue | 209.0788602 |
| TCID_50_ | CPE of Vero cells | Spike-B.1.351 *(IMx2)* +  Spike-B.1.351-NM *(IM)* | Day 138 | 65249 | TCID_50_**/**  100 mg tissue | 543.0292599 |
| TCID_50_ | CPE of Vero cells | Spike-B.1.351 *(IMx2)* +  Spike-B.1.351-NM *(IM)* | Day 138 | 65250 | TCID_50_**/**  100 mg tissue | 721.9679634 |
| TCID_50_ | CPE of Vero cells | Spike-B.1.351 *(IMx2)* +  Spike-B.1.351-NM *(IM)* | Day 138 | 65251 | TCID_50_**/**  100 mg tissue | 357.1024335 |
| TCID_50_ | CPE of Vero cells | Spike-B.1.351 *(IMx2)* +  Spike-B.1.351-NM *(IM)* | Day 138 | 65252 | TCID_50_**/**  100 mg tissue | 785.8032379 |
| TCID_50_ | CPE of Vero cells | Spike-B.1.351 *(IMx2)* +  Spike-B.1.351-NM *(IM)* | Day 138 | 65253 | TCID_50_**/**  100 mg tissue | 596.4083176 |
| TCID_50_ | CPE of Vero cells | Spike-B.1.351 *(IMx2)* +  Spike-B.1.351-NM *(IM)* | Day 138 | 65254 | TCID_50_**/**  100 mg tissue | 485.3846154 |
| TCID_50_ | CPE of Vero cells | Spike-B.1.351 *(IMx2)* +  Spike-B.1.351-NM *(IM)* | Day 138 | 65255 | TCID_50_**/**  100 mg tissue | 644.53524 |
| TCID_50_ | CPE of Vero cells | Spike-B.1.351 *(IMx2)* +  Spike-B.1.351-NM *(IM)* | Day 138 | 65256 | TCID_50_**/**  100 mg tissue | 751.1904762 |
| TCID_50_ | CPE of Vero cells | Buffer only *(IMx3)* | Day 138 | 65242 | TCID_50_**/**  100 mg tissue | 6755.888651 |
| TCID_50_ | CPE of Vero cells | Buffer only *(IMx3)* | Day 138 | 65243 | TCID_50_**/**  100 mg tissue | 52803.34728 |
| TCID_50_ | CPE of Vero cells | Buffer only *(IMx3)* | Day 138 | 65244 | TCID_50_**/**  100 mg tissue | 114727.2727 |
| TCID_50_ | CPE of Vero cells | Buffer only *(IMx3)* | Day 138 | 65245 | TCID_50_**/**  100 mg tissue | 6247.524752 |

S12 Table. Amount of infectious virus in nasal turbinates of mice 5 days post-intranasal infection with SARS-CoV-2 BA.5

| **Assay** | **Matrix** | **Treatment** | **Study Day** | **Animal No.** | **Units** | **Data** |
| --- | --- | --- | --- | --- | --- | --- |
| TCID_50_ | CPE of Vero cells | Spike-B.1.351*(IMx3)* | Day 138 | 65228 | TCID_50_**/**  100 mg tissue | 143.4090909 |
| TCID_50_ | CPE of Vero cells | Spike-B.1.351*(IMx3)* | Day 138 | 65230 | TCID_50_**/**  100 mg tissue | 156.5756824 |
| TCID_50_ | CPE of Vero cells | Spike-B.1.351*(IMx3)* | Day 138 | 65232 | TCID_50_**/**  100 mg tissue | 160.5597964 |
| TCID_50_ | CPE of Vero cells | Spike-B.1.351*(IMx3)* | Day 138 | 65233 | TCID_50_**/**  100 mg tissue | 214.6258503 |
| TCID_50_ | CPE of Vero cells | Spike-B.1.351*(IMx3)* | Day 138 | 65234 | TCID_50_**/**  100 mg tissue | 172.8767123 |
| TCID_50_ | CPE of Vero cells | Spike-B.1.351*(IMx3)* | Day 138 | 65235 | TCID_50_**/**  100 mg tissue | 145.0574713 |
| TCID_50_ | CPE of Vero cells | Spike-B.1.351 *(IMx2)* +  Spike-B.1.351-NM *(IM)* | Day 138 | 65249 | TCID_50_**/**  100 mg tissue | 79.67171717 |
| TCID_50_ | CPE of Vero cells | Spike-B.1.351 *(IMx2)* +  Spike-B.1.351-NM *(IM)* | Day 138 | 65250 | TCID_50_**/**  100 mg tissue | 102.7687296 |
| TCID_50_ | CPE of Vero cells | Spike-B.1.351 *(IMx2)* +  Spike-B.1.351-NM *(IM)* | Day 138 | 65251 | TCID_50_**/**  100 mg tissue | 132.5630252 |
| TCID_50_ | CPE of Vero cells | Spike-B.1.351 *(IMx2)* +  Spike-B.1.351-NM *(IM)* | Day 138 | 65252 | TCID_50_**/**  100 mg tissue | 138.0743982 |
| TCID_50_ | CPE of Vero cells | Spike-B.1.351 *(IMx2)* +  Spike-B.1.351-NM *(IM)* | Day 138 | 65253 | TCID_50_**/**  100 mg tissue | 165.1832461 |
| TCID_50_ | CPE of Vero cells | Spike-B.1.351 *(IMx2)* +  Spike-B.1.351-NM *(IM)* | Day 138 | 65254 | TCID_50_**/**  100 mg tissue | 133.970276 |
| TCID_50_ | CPE of Vero cells | Spike-B.1.351 *(IMx2)* +  Spike-B.1.351-NM *(IM)* | Day 138 | 65255 | TCID_50_**/**  100 mg tissue | 117.0686456 |
| TCID_50_ | CPE of Vero cells | Spike-B.1.351 *(IMx2)* +  Spike-B.1.351-NM *(IM)* | Day 138 | 65256 | TCID_50_**/**  100 mg tissue | 139.6017699 |
| TCID_50_ | CPE of Vero cells | Buffer only *(IMx3)* | Day 138 | 65242 | TCID_50_**/**  100 mg tissue | 3875.968992 |
| TCID_50_ | CPE of Vero cells | Buffer only *(IMx3)* | Day 138 | 65243 | TCID_50_**/**  100 mg tissue | 743.2273263 |
| TCID_50_ | CPE of Vero cells | Buffer only *(IMx3)* | Day 138 | 65244 | TCID_50_**/**  100 mg tissue | 7328.687573 |
| TCID_50_ | CPE of Vero cells | Buffer only *(IMx3)* | Day 138 | 65245 | TCID_50_**/**  100 mg tissue | 1427.60181 |

S13 Table. Amount of SARS-CoV-2 viral RNA in lungs of mice 5 days post-intranasal infection with SARS-CoV-2 BA.5

| **Assay** | **Matrix** | **Treatment** | **Study Day** | **Animal No.** | **Units** | **Data** |
| --- | --- | --- | --- | --- | --- | --- |
| qPCR | Ct value | Spike-B.1.351*(IMx3)* | Day 138 | 65228 | RNA copies **/** g tissue | 1316316.431 |
| qPCR | Ct value | Spike-B.1.351*(IMx3)* | Day 138 | 65230 | RNA copies **/** g tissue | 132157.8254 |
| qPCR | Ct value | Spike-B.1.351*(IMx3)* | Day 138 | 65232 | RNA copies **/** g tissue | 1443.688677 |
| qPCR | Ct value | Spike-B.1.351*(IMx3)* | Day 138 | 65233 | RNA copies **/** g tissue | 9958.055369 |
| qPCR | Ct value | Spike-B.1.351*(IMx3)* | Day 138 | 65234 | RNA copies **/** g tissue | 2663732.851 |
| qPCR | Ct value | Spike-B.1.351*(IMx3)* | Day 138 | 65235 | RNA copies **/** g tissue | 3664.138174 |
| qPCR | Ct value | Spike-B.1.351 *(IMx2)* +  Spike-B.1.351-NM *(IM)* | Day 138 | 65249 | RNA copies **/** g tissue | 13234225.51 |
| qPCR | Ct value | Spike-B.1.351 *(IMx2)* +  Spike-B.1.351-NM *(IM)* | Day 138 | 65250 | RNA copies **/** g tissue | 1193.181482 |
| qPCR | Ct value | Spike-B.1.351 *(IMx2)* +  Spike-B.1.351-NM *(IM)* | Day 138 | 65251 | RNA copies **/** g tissue | 6563984.114 |
| qPCR | Ct value | Spike-B.1.351 *(IMx2)* +  Spike-B.1.351-NM *(IM)* | Day 138 | 65252 | RNA copies **/** g tissue | 918511.4513 |
| qPCR | Ct value | Spike-B.1.351 *(IMx2)* +  Spike-B.1.351-NM *(IM)* | Day 138 | 65253 | RNA copies **/** g tissue | 31378011.32 |
| qPCR | Ct value | Spike-B.1.351 *(IMx2)* +  Spike-B.1.351-NM *(IM)* | Day 138 | 65254 | RNA copies **/** g tissue | 193592256.7 |
| qPCR | Ct value | Spike-B.1.351 *(IMx2)* +  Spike-B.1.351-NM *(IM)* | Day 138 | 65255 | RNA copies **/** g tissue | 135141.5229 |
| qPCR | Ct value | Spike-B.1.351 *(IMx2)* +  Spike-B.1.351-NM *(IM)* | Day 138 | 65256 | RNA copies **/** g tissue | 8447.740324 |
| qPCR | Ct value | Buffer only *(IMx3)* | Day 138 | 65242 | RNA copies **/** g tissue | 2.06012E+11 |
| qPCR | Ct value | Buffer only *(IMx3)* | Day 138 | 65243 | RNA copies **/** g tissue | 2.84659E+11 |
| qPCR | Ct value | Buffer only *(IMx3)* | Day 138 | 65244 | RNA copies **/** g tissue | 42411619559 |
| qPCR | Ct value | Buffer only *(IMx3)* | Day 138 | 65245 | RNA copies **/** g tissue | 81015726409 |

S14 Table. Amount of SARS-CoV-2 viral RNA in nasal turbinates of mice 5 days post-intranasal infection with SARS-CoV-2 BA.5

| **Assay** | **Matrix** | **Treatment** | **Study Day** | **Animal No.** | **Units** | **Data** |
| --- | --- | --- | --- | --- | --- | --- |
| qPCR | Ct value | Spike-B.1.351*(IMx3)* | Day 138 | 65228 | RNA copies **/** g tissue | 293689.012 |
| qPCR | Ct value | Spike-B.1.351*(IMx3)* | Day 138 | 65230 | RNA copies **/** g tissue | 261181.1759 |
| qPCR | Ct value | Spike-B.1.351*(IMx3)* | Day 138 | 65232 | RNA copies **/** g tissue | 26748.26561 |
| qPCR | Ct value | Spike-B.1.351*(IMx3)* | Day 138 | 65233 | RNA copies **/** g tissue | 165613.8778 |
| qPCR | Ct value | Spike-B.1.351*(IMx3)* | Day 138 | 65234 | RNA copies **/** g tissue | 1911125.527 |
| qPCR | Ct value | Spike-B.1.351*(IMx3)* | Day 138 | 65235 | RNA copies **/** g tissue | 215182.6672 |
| qPCR | Ct value | Spike-B.1.351 *(IMx2)* +  Spike-B.1.351 +NM *(IM)* | Day 138 | 65249 | RNA copies **/** g tissue | 432914.4706 |
| qPCR | Ct value | Spike-B.1.351 *(IMx2)* +  Spike-B.1.351-NM *(IM)* | Day 138 | 65250 | RNA copies **/** g tissue | 1161787.076 |
| qPCR | Ct value | Spike-B.1.351 *(IMx2)* +  Spike-B.1.351-NM *(IM)* | Day 138 | 65251 | RNA copies **/** g tissue | 72722.63498 |
| qPCR | Ct value | Spike-B.1.351 *(IMx2)* +  Spike-B.1.351-NM *(IM)* | Day 138 | 65252 | RNA copies **/** g tissue | 236440.0367 |
| qPCR | Ct value | Spike-B.1.351 *(IMx2)* +  Spike-B.1.351-NM *(IM)* | Day 138 | 65253 | RNA copies **/** g tissue | 1277195.675 |
| qPCR | Ct value | Spike-B.1.351 *(IMx2)* +  Spike-B.1.351-NM *(IM)* | Day 138 | 65254 | RNA copies **/** g tissue | 813693.2089 |
| qPCR | Ct value | Spike-B.1.351 *(IMx2)* +  Spike-B.1.351-NM *(IM)* | Day 138 | 65255 | RNA copies **/** g tissue | 880032.9716 |
| qPCR | Ct value | Spike-B.1.351 *(IMx2)* +  Spike-B.1.351-NM *(IM)* | Day 138 | 65256 | RNA copies **/** g tissue | 351859.3832 |
| qPCR | Ct value | Buffer only *(IMx3)* | Day 138 | 65242 | RNA copies **/** g tissue | 1995455.227 |
| qPCR | Ct value | Buffer only *(IMx3)* | Day 138 | 65243 | RNA copies **/** g tissue | 653554269.3 |
| qPCR | Ct value | Buffer only *(IMx3)* | Day 138 | 65244 | RNA copies **/** g tissue | 320565850 |
| qPCR | Ct value | Buffer only *(IMx3)* | Day 138 | 65245 | RNA copies **/** g tissue | 6542708.64 |

**S15 Table. Individual mouse lung pathology scores following SARS-CoV-2 infection**

| **Treatment** | **Animal No.** | **Lung Pathology Scoring** | |
| --- | --- | --- | --- |
|  |  | **Inflammatory Cell Infiltrates** | **Fibrosis** |
| Spike-B.1.351 *(IMx3)* | 65228 | 9.0 | 10.8 |
| Spike-B.1.351 *(IMx3)* | 65230 | 7.0 | 9.0 |
| Spike-B.1.351 *(IMx3)* | 65232 | 10.0 | 10.8 |
| Spike-B.1.351 *(IMx3)* | 65233 | 13.0 | 10.8 |
| Spike-B.1.351 *(IMx3)* | 65234 | 18.0 | 10.0 |
| Spike-B.1.351 *(IMx3)* | 65235 | 9.0 | 11.5 |
| Spike-B.1.351 *(IMx2)* +  Spike-B.1.351-NM *(IM)* | 65249 | 13.0 | 10.8 |
| Spike-B.1.351 *(IMx2)* +  Spike-B.1.351-NM *(IM)* | 65250 | 9.0 | 9.0 |
| Spike-B.1.351 *(IMx2)* +  Spike-B.1.351-NM *(IM)* | 65251 | 14.0 | 10.8 |
| Spike-B.1.351 *(IMx2)* +  Spike-B.1.351-NM *(IM)* | 65252 | 10.0 | 10.8 |
| Spike-B.1.351 *(IMx2)* +  Spike-B.1.351-NM *(IM)* | 65253 | 11.0 | 10.0 |
| Spike-B.1.351 *(IMx2)* +  Spike-B.1.351-NM *(IM)* | 65254 | 11.0 | 11.5 |
| Spike-B.1.351 *(IMx2)* +  Spike-B.1.351-NM *(IM)* | 65255 | 12.0 | 8.0 |
| Spike-B.1.351 *(IMx2)* +  Spike-B.1.351-NM *(IM)* | 65256 | 11.0 | 9.8 |
| Buffer only *(IMx3)* | 65242 | 15.6 | 17.4 |
| Buffer only *(IMx3)* | 65243 | 19.0 | 17.7 |
| Buffer only *(IMx3)* | 65244 | 19.2 | 19.8 |
| Buffer only *(IMx3)* | 65245 | 17.4 | 18.2 |

**S16 Table. Mouse sera neutralization titers following SARS-CoV-2 BA.5 Infection**

| **Treatment** | **Animal No.** | **Mouse sera ID_50_ Titers** | | | | | | |
| --- | --- | --- | --- | --- | --- | --- | --- | --- |
|  |  | **Wuhan-Hu-1**  **(D614G)** | **Beta B.1.351** | **BA.1** | **BA.2** | **BA.2.12.1** | **BA.5** | **XBB1.5** |
| Spike-B.1.351 *(IMx3)* | 65228 | 18834.6412 | 57902.68 | 1775.072 | 5237.497 | 9518.611 | 10131.08 | 1 |
| Spike-B.1.351 *(IMx3)* | 65230 | 24769.7172 | 32577.3 | 975.974 | 1850.3692 | 1405.009 | 8883.85 | 2039.83 |
| Spike-B.1.351 *(IMx3)* | 65232 | 5139.27985 | 5039.522 | 3105.042 | 295.7585 | 349.0365 | 1 | 1 |
| Spike-B.1.351 *(IMx3)* | 65233 | 94594.6222 | 287638.3 | 15525.49 | 3797.995 | 3251.872 | 12565.54 | 467.5 |
| Spike-B.1.351 *(IMx3)* | 65234 | 14263.1209 | 58350.66 | 4040.032 | 6407.258 | 5152.077 | 7199.201 | 1 |
| Spike-B.1.351 *(IMx3)* | 65235 | 5375.26482 | 4691.189 | 1157.127 | 560.4477 | 1 | 1 | 1 |
| Spike-B.1.351 *(IMx2)* +  Spike-B.1.351-NM *(IM)* | 65249 | 24485.9867 | 62155.73 | 2495.249 | 1081.067 | 398.7108 | 2486.916 | 563.883 |
| Spike-B.1.351 *(IMx2)* +  Spike-B.1.351-NM *(IM)* | 65250 | 74852.4775 | 103666.5 | 1421.334 | 15133.72 | 16022.75 | 59937.38 | 1 |
| Spike-B.1.351 *(IMx2)* +  Spike-B.1.351-NM *(IM)* | 65251 | 40178.8555 | 124480.3 | 9093.706 | 3111.332 | 2033.332 | 8091.17 | 1 |
| Spike-B.1.351 *(IMx2)* +  Spike-B.1.351-NM *(IM)* | 65252 | 13769.0818 | 59381.79 | 1731.887 | 1080.039 | 708.6641 | 4958.043 | 1 |
| Spike-B.1.351 *(IMx2)* +  Spike-B.1.351-NM *(IM)* | 65253 | 76093.6646 | 103908.6 | 2849.021 | 1264.955 | 1052.422 | 868.5389 | 1 |
| Spike-B.1.351 *(IMx2)* +  Spike-B.1.351-NM *(IM)* | 65254 | 30325.8674 | 19839.35 | 1777.159 | 867.431 | 622.7478 | 1582.36 | 1 |
| Spike-B.1.351 *(IMx2)* +  Spike-B.1.351-NM *(IM)* | 65255 | 179121.557 | 401234 | 16115.05 | 9906.981 | 7132.647 | 7293.971 | 1 |
| Spike-B.1.351 *(IMx2)* +  Spike-B.1.351-NM *(IM)* | 65256 | 81238.2809 | 67563.12 | 6725.48 | 1667.689 | 3150.98 | 1760.808 | 1 |
| Buffer only *(IMx3)* | 65242 | 1 | 1 | 1 | 1 | 1 | 1 | 1 |
| Buffer only *(IMx3)* | 65243 | 1 | 1 | 1 | 1 | 1 | 465 | 1 |
| Buffer only *(IMx3)* | 65244 | 1 | 1 | 1 | 283 | 532 | 663 | 1 |
