# Supplementary material for: Comparison of a SARS-CoV-2 mRNA booster immunization containing additional antigens to a spike-based mRNA vaccine against Omicron BA.5 infection in hACE2 mice
Source: PLoS One. 2024 Dec 3;19(12):e0314061. doi: 10.1371/journal.pone.0314061 (PMC11614295; doi:10.1371/journal.pone.0314061)
Supplement: Attachment — Submitted filename: Response_to_Reviewers.docx [file pone.0314061.s002.docx]

July 19, 2024

Dear Dr. Raju

We would like to thank the reviewers and PLoS One editorial team for giving us the chance to resubmit our revised manuscript. We thank the reviewers for their thoughtful questions and comments. We have edited the manuscript accordingly and have included below a point-by-point response to each of their questions.

Best wishes,

Rob Kozak, PhD, FCCM

**Response to reviewers**

Reviewer #1 Comments and Author Responses (**written in bold**).

 The authors attempted to compare the immunogenicity and protective efficacy of a booster dose of mRNA encoding the S protein of the Beta variant alone or in combination with Wuhan M+N. Overall, the study design is sound and rational, and the strategy to answer the research question is scientifically valid. However, minor additional explanations are required:

**We thank Reviewer #1 for their overall positive comments on the manuscript.**

1. The mRNA dose used in this study is relatively high (5-10 µg) in mice compared to other studies that used 1-2 µg per dose. Please discuss and compare the vaccine dose with those used in other studies. There is a concern that the high mRNA dose (5 or 10 µg) used in this study may obscure differences between treatment groups, as it could reach the plateau of antibody titers.

**While the antibody plateau effect from high mRNA doses is a concern in some contexts, it is not universally observed across all studies and vaccine candidates. In the studies referenced below, the authors have shown that immune response to higher mRNA doses can vary, and incremental benefits can still be observed even at doses that are considered high. For example, in Corbett et al., nonhuman primates received 10 or 100 mcg of mRNA-1273. The higher doses of the mRNA-1273 vaccine did not show a simple plateau in antibody responses, and differences in antibody responses were still detected at the higher dose (Corbett et al., 2020, N Engl J Med, DOI: 10.1056/NEJMoa2024671). Additionally, in the phase 1 trial reporting of a dose-escalation of mRNA-1273, participants received two vaccine doses of either 25 mcg or 100 mcg of mRNA administered 28 days apart. Anderson et al showed that virus neutralization was dose-dependent, with responses to the 100-mcg dose higher than responses to the 25-mcg dose, and this neutralizing activity remained high through 4 weeks after the administration of the second dose in all the subgroups (Anderson et al., 2020, N Engl J Med, DOI: 10.1056/NEJMoa2028436). In Vogel et al., antibody responses characterized in BALB/c mice after a single intramuscular injection of 0.2-, 1- or 5-mcg show that 50% pseudovirus-neutralization titers were dose-dependent and were greater for the 5-mcg dose compared with the 1- or 0.2-mcg dose at 28 days post-vaccination. Furthermore, both binding and 50% pseudovirus-neutralization titers showed similar dose-dependent responses in rhesus macaques injected with 30 mcg or 100 mcg with geometric mean titers (GMTs) nearly double for the 100-mcg dose drawn 7 days after dose 2. By 21 days post-dose 2, titers for the 100-mcg dose remained at more than double the GMTs for the 30-mcg dose (Vogel et al., 2021, Nature, DOI: 10.1038/s41586-021-03275-y). Results from these studies demonstrate that higher doses continue to elicit robust antibody responses and that the immune system's response can still be dose-dependent even at higher mRNA doses without clear evidence of a plateau, providing a more nuanced understanding of the dose-response relationship.**

**Nevertheless, when we reduced the amount of the spike in the heterologous spike-NM booster to 5 mcg to keep total dose of the spike-NM mRNA vaccine at 10 mcg, we did not observe any biological effect on the protection against the BA.5 challenge or neutralizing responses, likely because all vaccinated mice received the same 10 μg dose of spike for their first two immunizations. We have included the following statement on lines 539-551 to discuss how the high mRNA dose (5 or 10 µg) used in this study is unlikely obscuring differences between treatment groups, citing the studies above to describe that the more nuanced relationship with mRNA dose-responses: “The mRNA vaccine doses used in this study (5-10 µg) may be considered high relative to other studies that use 1-2 µg per dose (46,47), however, vaccines containing 30 µg of mRNA have proven effective in mice without evidence of antibody-dependent enhancement of infection by virus-specific antibodies (48). Furthermore, incremental benefits to immune responses can still be observed even at doses that are considered high (49-51), providing a more nuanced understanding of the mRNA dose-response relationship. The amount of spike in the heterologous spike-NM booster was decreased to 5 μg to maintain the overall dose of the spike-NM mRNA vaccine at 10 μg. Here, we demonstrate that this variation in the amount of spike in the third dose had no biological effect on protection against the BA.5 challenge or neutralizing responses, likely because all vaccinated mice received the same 10 μg dose of spike for their first two immunizations. Despite containing half the amount of spike mRNA as the monovalent spike-only, the spike-NM booster used in this study induced potent nAb titers that were comparable or non-inferior to the spike booster alone** **including nAbs to a broad range of Omicron spike antigenic variants.”**

2. In writing, the authors should emphasize that the amount of S-mRNA in the spike-only group is double that in the multiantigen group. Thus, the efficacy contributed by anti-S antibodies may not be directly comparable.

**We thank Reviewer #1 for their comment. The efficacy of the anti-S antibodies may not be directly comparable between the two vaccine groups as the group that received the heterologous spike-NM booster received half the amount of spike mRNA in their third dose (5 mcg) compared with the spike-only group that received 10 mcg of spike mRNA in their third dose. For the purpose of this study, we focused on making sure the heterologous booster was not inferior to the standard homologous spike-only booster and the results of the endpoint pseudovirus neutralizing titers confirmed this showing similar neutralization of the homologous Beta (B.1.351) strain (Figure 7). In lines 544-551 of the discussion section, we have included the text “The amount of spike in the heterologous spike-NM booster was decreased to 5 μg to maintain the overall dose of the spike-NM mRNA vaccine at 10 μg. Here, we demonstrate that this variation in the amount of spike in the third dose had no biological effect on protection against the BA.5 challenge or neutralizing responses, likely because all vaccinated mice received the same 10 μg dose of spike for their first two immunizations. Despite containing half the amount of spike mRNA as the monovalent spike-only, the spike-NM booster used in this study induced potent nAb titers that were comparable or non-inferior to the spike booster alone, including nAbs to a broad range of Omicron spike antigenic variants.”**

3. Why is there no neutralization titer (Nt) data for the Beta variant in Figures 3A and 3B?

**All data presenting immune responses to the Beta spike variant is shown in Figure 2, Immunogenicity of Spike B.1.351 in K18-hACE2 mice. In Figure 2C, serum neutralizing antibody titers to Beta spike are shown at 3 weeks and 8 weeks following the second vaccination. The text describing these results are under subheading “Spike B.1.351 vaccination induces strong spike-specific cell-mediated and humoral immunity in K18-hACE2 mice.”**

**In Figure 3, Pseudovirus neutralization of SARS-CoV-2 VOCs from vaccinated mice sera, we present the cross-neutralization against Wuhan-Hu-1 (D614G), and Omicron (BA.1, BA.2, BA.2.12.1, BA.5, and XBB1.5) spike variants that were not included in the vaccine formulation. The text describing these results are under subheading “Spike B.1.351 only vaccine does not induce broadly neutralizing antibody titers in K18-hACE2 mice.”**

Reviewer #2 Comments and Author Responses (**written in bold**).

The manuscript of J. Hurst et al., ‘Comparison of a SARS-CoV-2 mRNA booster immunization containing additional antigens to a spike-based mRNA vaccine against Omicron BA.5 infection in hACE2 mice,’ is dedicated to assessing the potential value of SARS-CoV-2 nucleoprotein (N) and membrane (M) proteins in protection against new variants of SARS-CoV-2 infection.

The authors postulated that the vaccines expressing more conservative SARS-CoV-2 antigens, such as nucleocapsid and membrane proteins, might induce robust T cell immunity with a high frequency of cytokine-producing CD8+ T cells and be associated with a reduction of disease severity and/or a broader cross-neutralizing immune response. The article is meticulously crafted, comprehensively describing the methods employed, key results, and a thorough review of the available literature.

**We thank Reviewer #2 for their positive comments on the manuscript.**

Overall, the results are significant, indicating that the vaccine expressing anti-N and anti-M proteins is not associated with better spike-specific cell-mediated and humoral immunity (as expected) but also does not improve the prevention of heterologous SARS-CoV-2 infection in the transgenic mice challenge model. The N- and M-containing candidate has a lower impact on viral shedding than the monovalent anti-S comparator, induces comparable protection from severe lung pathology, and does not increase the breadth of neutralizing antibodies against heterologous SARS-CoV-2 strains.
The study results indicate that including more conservative epitopes in the vaccine composition does not translate into better protection or immunogenicity benefits in mice models. The study results indicate that including more conservative epitopes in the vaccine composition does not translate into better protection or immunogenicity benefits in mice models.

In the discussion section, the authors try to justify the inclusion of N and M antigens, stating that they provide equal efficacy as a spike-only booster. However, the dose of mRNA used in the study (10 mcg) is relatively high for a booster, and 5 and 10 mcg can induce a similar immune response and be associated with a similar protection. As such, the hypothesis that the expression of anti-N and anti-M antibodies provides additional benefits is not supported by the data. Overall, the discussion overstated the value of the inclusion of N and M antigens, and the data do not support the conclusion.

**We thank Reviewer #2 for recognizing that the immunogenicity testing in our study has limitations. Unfortunately, not all Omicron subvariant strains were available for use in our high-containment facility at the time of testing and therefore we were only able to measure the neutralization capacity of post-vaccination serum by spiked-pseudotyped lentivirus neutralization assays and not live virus assays. As a result, only spike-specific antibodies were specifically measured,** **and we show that neutralization against the matched pseudovirus harbouring the Beta B.1.351 spike was not different between mice that received the homologous Beta spike-only or heterologous spike-NM boosters (Figure 7). When measuring endpoint serum against heterologous pseudoviruses expressing spike proteins from Omicron subvariants, we also saw no difference in the neutralization response between the vaccinated groups, although neither group showed significantly higher neutralization of Omicron variants than control mice (Figure 7). However, endpoint neutralization titers against the Wuhan-Hu-1 strain was significantly greater for the spike-NM vaccinated mice when compared to control mice, but not for the mice that received spike-only (Figure 7). While we did not show superior protection against Omicron BA.5 challenge or spreading, we did demonstrate that reducing the amount of spike from 10 μg to 5 μg and adding N and M to a heterologous booster dose does not alter the neutralization effects from spike-specific neutralizing antibodies, and protection against Omicron BA.5 infection is comparable with near complete inhibition of infectious virus shedding in oral swabs and reduced viral burdens in both lung and nasal tissues following BA.5 challenge.**

**Although anti-N and anti-M antibodies were not specifically measured in the spike-pseudotyped neutralization assays, we speculate they may have important roles in disease modulation outside of neutralizing antibodies and we have included some literature references that describe their function outside of neutralization. For example, on lines 608-613 we referenced Díez et al who demonstrate that antibodies against the N protein in hyperimmune human convalescent plasma induced powerful antibody-dependent cellular phagocytosis (ADCP) and antibody-dependent cellular cytotoxicity (ADCC) activities in antigen-dependent Fc function experiments. The ADCC and ADCP are mechanisms for antigen-dependent antibodies through which virus-infected cells are targeted for destruction or elimination primarily through FcγRIIIa expressed on natural killer cells for ADCC and by monocytes, macrophages, neutrophils, and dendritic cells via FcγRIIa (CD32a), FcγRI (CD64), and FcγRIIIa (CD16a) for ADCP. Thus,** **the role of antibodies against SARS-CoV-2 N protein could facilitate antigen uptake and presentation and may supplement neutralizing activity by enabling non-neutralizing antibodies or antibodies with poor-neutralizing capacity to block or clear infection through S-protein independent mechanisms (Díez et al., 2022, JID, DOI: 10.1093/infdis/jiab540). We also refer to work by López-Muñoz et al on lines 613-615 that supports this, showing that N protein is abundantly present on the surface of infected cells and can be targeted by Fc-expressing innate immune cells (López-Muñoz et al., 2022, Sci Adv, eabp9770). While we did not directly measure any effect of anti-N and anti-M antibodies, incorporating N and M in a SARS-CoV-2 vaccine could lead to improved neutralization and/or viral clearance. Although we did not see improved protection in mice for the spike-NM booster compared with the spike-only booster, our study was limited by a low number of mice and may not have caught their contribution. Future work could consider testing just the N and M chimeric mRNA alone as a separate vaccine group, however, we do not expect N and M alone to outperform a formulation that contains spike, and thus, this was not included in our study. To determine if adding N and M led to improved neutralization, we discuss on lines 620-623 “to test the role of N or M-specific antibodies from vaccinated mice in this study, mouse sera could be depleted of antibodies against spike and the RBD before testing the neutralization capacity for the remaining antibodies and the effect on complement deposition.” This was not completed in our study as all mouse sera was utilized for the pseudovirus neutralization assays.**

Some important information is missing in the Methods section:
1. Immunogen design is not presented (stabilized vs. native spike?)

**The spike immunogen encoded in the mRNA vaccines are full-length native spike from VOC B.1.351, without proline substitutions for perfusion stabilization. We have added this information to our methods under the vaccine formulation section in lines 99-100 “The spike mRNA vaccine used in these studies encode the full-length spike from Beta B.1.351 that does not contain the 2P stabilization.”**

2. Is it modified or non-modified mRNA?

**The mRNA in the vaccine is modified mRNA using N1-methyl-pseudouridine. We have added this information to our methods under the vaccine formulation section, lines 102-104 “All mRNAs used were modified mRNA using N1-methyl-pseudouridine and formulated in lipid nanoparticles as described previously (40).”**

3. How was the expression of all target proteins controlled/verified? Was a potency assay used? Without measuring anti-N and anti-M antibodies, how did the authors confirm the sufficient level of expression of these antigens?

**Expression of the spike protein can be verified through the production of neutralizing antibody responses to the matched SARS-CoV-2 Beta (B.1.351) pseudovirus following spike-B.1.351 vaccinations, as seen in Figure 2C. Furthermore, spike-B.1.351 vaccinations induced 838.75 ± 220.37 (means ± SEM) IFN-g spot-forming units (SFU) per 2.5 x 10^5^ splenocytes by ELISPOT (Figure 2A), and both CD4^+^ and CD8^+^ T cells from vaccinated mice had increased percentages of IFN-g, TNF-a, and IL-2–producing cells following stimulation with Beta spike (Figure 2B). These results indicate a strong induction of a TH1 response to the spike protein, confirming expression of the spike mRNA.**

**Expression of N and M target proteins were verified in a potency assay as an essential step of mRNA vaccine production at Providence Therapeutics, including measuring the concentration of mRNAs before encapsulation. Attached below is an SDS-PAGE gel of Hela cell lysates following *in-vitro* translation of the NM mRNA construct that encodes the fusion chimerical protein of N and M.** **This figure illustrates expression of the NM mRNA after transfection into the cells, as the chimera NM protein migrated to the expected molecular weight (~41 kDa) representing the fusion of the N (Theoretical M.W. 28.1 kDa) and M (13.6 kDa) fragments.**

4. How did the authors select the dose level (10 mcg) for the mice experiments? If the intention is to demonstrate the value of N and M antigen inclusion, what was the rationale for reducing the S dose to 5 mcg?

**We previously compared the dose of our spike mRNA vaccine at 1 mcg and 10 mcg in C57BL/6 mice, and 4 mcg and 10 mcg in Balb/c mice (Liu et al., 2022, Sci. Adv. 8, eabj9815), and found that 10 mcg induced the highest nAb titers against Wuhan-Hu-1 and VOCs. We therefore used spike only mRNA vaccine at 10 mcg dose. We have included the following text to lines 121-123: “Previous work comparing spike mRNA vaccine doses in C57BL/6 and BALB/c mice found that a 10 μg dose elicited the highest nAb titers against Wuhan-Hu-1 and VOCs (40), and thus, a 10 μg dose of the spike-only mRNA vaccine was utilized for this study.” As a first step to test our multi-antigenic COVID-19 mRNA vaccine, we decided to make a 1:1 mass ratio mixture of the spike mRNA and the N/M chimera mRNA. Thus, we reduced the amount of the spike in the heterologous spike-NM booster to 5 mcg to keep total dose of the spike-NM mRNA vaccine at 10 mcg, which was equal to the spike only mRNA vaccine used in comparison group. We have included this information on lines 140-141, stating “The amount of spike in the heterologous spike-NM booster was decreased to 5 μg to maintain the overall dose of the spike-NM mRNA vaccine at 10 μg.” Overall, all vaccinated mice received the same 10-mcg dose of spike for their first two immunizations, and we showed that the slight reduction in the amount of spike in the third booster did not have any biological effect on protection from BA.5 challenge (Figures 5-6) or neutralizing responses (Figure 7).**

5. Since a separate drug substance with N and M genes was produced, do the authors consider testing an anti-N/anti-M vaccine without spike protein to assess the stand-alone value of such vaccine composition?

**The multiantigenic booster approach we describe in our manuscript incorporates additional more conserved antigens relative to spike-only vaccines. We had hypothesized that this approach would induce more broadly protective responses and provide an advantage over currently available vaccines that target spike-only. Our rationale for combining the mRNAs containing spike only and the chimeric mRNA containing N and M targets and not comparing against the stand-alone NM-containing mRNA formulation was based on work by O’Donnell KL et al and Dangi et al referenced within lines 582-589. These authors report the efficacies of their multivalent vaccines that co-express S and N on vesicular stomatitis virus (VSV) or adenovirus and found that the combination of S and N antigens broadens vaccine protection to the brain and lungs compared to the S only vaccine that protects against the lungs alone, concluding that addition of the N protein indeed improved protective efficacy (O’Donnell et al., 2022, Front Immunol; Dangi et al., 2021, Cell Reports). Since neutralization of SARS-CoV-2 is primarily driven by immune responses against the spike antigen (or receptor binding domain), a formulation that does not express spike will unlikely be effective by itself, and thus, a multivalent vaccine expressing N and M proteins is best suited in synergy with immunity directed against the spike protein.**
